# Relation of Pain to Swallowing

**Published:** 1894-02

**Authors:** A. McNeil

**Affiliations:** San Francisco, Cal.


### RELATION OF PAIN TO SWALLOWING.

San Francisco, Cal., November 7th, 1893.

Editor of The Homœopathic Physician :—In the November number of The Homœopathic Physician you give me credit for "The Relation of Pain to Swallowing." It is a translation I made from Bœnninghausen's *Aphorismen des Hippocrates.*

Please correct this and oblige, fraternally,

A. McNeil.

